# Perceived importance, self-rated performance satisfaction, learning stress, and practicum experiences among nursing students: A cross-sectional study with supplementary open-ended responses

**DOI:** 10.1371/journal.pone.0361978

**Published:** 2026-11-05

**Authors:** Hsiu-Wen Tsai, Shu-Ching Chen

**Affiliations:** 1 Department of Nursing, Hsin-Sheng Junior College of Medical Care and Management, Taoyuan, Taiwan; 2 School of Nursing and Center for Health and Geriatric Care Research and Development, College of Nursing, Chang Gung University of Science and Technology, Taoyuan, Taiwan; 3 Department of Radiation Oncology and Proton and Radiation Therapy Center, Chang Gung Medical Foundation, Linkou Chang Gung Memorial Hospital, Taoyuan, Taiwan; 4 School of Nursing, College of Medicine, Chang Gung University, Taoyuan, Taiwan; AIIMS: All India Institute of Medical Sciences, INDIA

## Abstract

This cross-sectional study examined nursing students’ perceived importance and self-rated performance satisfaction regarding fundamental nursing practicum competencies, explored learning-stress scores across academic-achievement categories and psychological-counseling history, and descriptively summarized written practicum experiences. All 730 eligible third-year nursing students were invited; 28 did not participate, 702 returned signed consent forms and questionnaires, and 3 incomplete questionnaires were excluded, leaving 699 participants. Importance–performance analysis (IPA) used sample grand means (importance, 4.679; performance satisfaction, 4.503) as crosshairs. Paired importance–performance gaps and 95% confidence intervals were calculated, and 5,000 bootstrap samples assessed coordinate uncertainty and quadrant stability. Exploratory grade-group and counseling-history comparisons used analysis of variance and independent-samples t tests, respectively, with Benjamini–Hochberg false-discovery-rate correction. Exploratory adjusted linear models used heteroskedasticity-consistent (HC3) standard errors. The Core Competence in Fundamental Nursing Practicum Scale importance and satisfaction scores showed very high internal consistency (alpha/omega = .987/.987 and .985/.986, respectively), potentially indicating item redundancy. The nursing-process domain received high absolute ratings but fell below both sample grand means; its importance–performance gap was 0.229 (95% CI, 0.179–0.280). Communication and biomedical-science quadrant classifications were unstable across bootstrap samples, indicating sensitivity to the selected crosshairs. Exploratory analyses using the adapted learning-stress questionnaire showed higher reported stress scores among students in the lowest academic-achievement category. However, maximum-likelihood and ordinal diagonally weighted least-squares confirmatory factor analyses did not support the prespecified two-factor structure, and within-cluster dependence could not be modeled. Counseling-history comparisons were descriptive and did not evaluate counseling effectiveness. All 699 participants answered both open-ended questions; after non-substantive entries were excluded from thematic assignment, 217 non-mutually-exclusive segment-to-theme assignments remained. These findings describe students’ perceptions and exploratory associations rather than observed competence, validated stress constructs, or causal effects.

## Introduction

The rapid evolution of healthcare systems has brought increased expectations for nursing students to demonstrate high levels of clinical competence before graduation [1]. Nursing education should ensure that students acquire theoretical knowledge and develop practical competencies through effective clinical and fundamental nursing practicums. The fundamental nursing practicum serves as the cornerstone of professional formation, providing opportunities for students to integrate the knowledge, skills, and attitudes essential for safe and effective patient care [2]. Gaps between theoretical knowledge and practical competencies can diminish the effectiveness of learning and weaken students’ preparedness for clinical practice [3]. The practicum bridges this gap between theory and practice, enabling students to develop their professional identity, critical thinking skills, and nursing care skills necessary for clinical excellence [4]. However, discrepancies exist between the competencies emphasized by educators and those that students perceive as important [5,6].

Assessing students’ perceived importance of practicum competencies alongside their self-rated performance satisfaction can help educators identify discrepancies across competency domains [7]. Importance–performance analysis (IPA) provides a descriptive map of relative perceived priorities but does not assess observed competence or establish teaching effectiveness [8]. Classroom and clinical-practicum stress may also influence students’ learning experiences and well-being [9,10]. Examining stress scores in relation to prior academic achievement and psychological-counseling history may identify patterns for further investigation; however, cross-sectional associations cannot establish directionality, causality, or counseling effectiveness [11,12]. This study aimed to: (1) describe students’ perceived importance and self-rated performance satisfaction across fundamental nursing practicum competency domains; (2) examine their relative IPA positions, paired gaps, and classification uncertainty; (3) explore classroom, practicum, and overall stress-score patterns across prior academic-average categories and psychological-counseling history; (4) estimate exploratory covariate-adjusted associations with these stress scores; and (5) descriptively summarize supplementary written practicum comments.

### Core competence and perceived performance in the fundamental nursing practicum

Previous studies have examined nursing students’ perceived competencies and self-ratings. Chang et al. [13] reported differences in perceived importance-performance gaps across placement settings. Other studies have identified perceived curricular priorities and discrepancies between perceived importance and training exposure [14,15]. Longitudinal work suggests that perceived stress, coping, and clinical competence ratings may change across practicum stages [16], whereas cross-sectional findings indicate that perceived competence varies with supervision, learning resources, and self-efficacy [17]. These findings concern perceptions or associations and should not be interpreted as objective evidence that clinical exposure causes competence development.

### Importance-performance analysis in nursing education

IPA has been used to describe the relative position of expected or perceived importance and self-rated performance in curriculum evaluation [18]. Its high-importance/low-performance region may suggest topics for further review, but quadrant placement depends on the selected crosshairs and does not itself establish educational need, curricular effectiveness, or observed competence. Therefore, IPA findings should be interpreted alongside absolute scale scores, uncertainty, and other evidence [14,19].

### Learning stress among nursing students

Nursing students report stress related to academic workload, clinical responsibilities, patient care, and evaluation [20]. Cross-sectional studies have found associations between lower grade-point averages and higher perceived stress [20], but directionality cannot be determined. Students who receive psychological counseling may differ systematically from those who do not, creating confounding by indication. Consequently, counseling-group comparisons should be interpreted as descriptive associations rather than evidence of counseling effectiveness [21,22].

## Materials and methods

### Study design, setting, and participants

This cross-sectional observational study used quantitative questionnaires supplemented by open-ended responses. An attempted census of third-year students in the Department of Nursing was conducted from October to November 2023 at Hsin-Sheng Junior College of Medical Care and Management in northern Taiwan; thus, the study involved one academic department. Students had completed their fundamental nursing practicum during the summer transition from the second to third year. The third-year cohort comprised 16 classes, with four classes undertaking each successive three-week off-campus practicum rotation. Students were distributed approximately evenly across 29 medical-surgical wards or practicum units at 22 accredited teaching-hospital campuses located across northern and central Taiwan. Across all rotations, 29 unique school-employed clinical instructors provided supervision. Within each practicum unit, one instructor supervised seven students per rotation and remained assigned to that unit while a new group of students rotated every three weeks. Eligible participants were third-year students who had completed the fundamental nursing practicum. No exclusion criteria were prespecified. Students who did not wish to participate did not return a signed consent form and were not enrolled.

The manuscript was prepared in accordance with the STROBE reporting guidance for cross-sectional observational studies. Students were nested within 16 classes and were additionally grouped by three-week rotation, practicum unit, teaching-hospital campus, and clinical instructor. However, the questionnaire did not collect participant-level identifiers for class, rotation, instructor, hospital, ward, or practicum unit. Consequently, individual participants could not be linked to specific clusters, and intraclass correlation coefficients, cluster-robust standard errors, and multilevel models could not be estimated.

### Data collection

The principal investigator and a research assistant recruited participants at the school; course and practicum instructors were not involved in recruitment. All 730 eligible students were invited to participate. Participation was voluntary and had no effect on course grades, practicum evaluations, or other academic assessments. Twenty-eight students did not return a signed consent form and therefore were not enrolled. The remaining 702 students returned signed written informed-consent forms and submitted questionnaires. Participants younger than 18 years took the consent documents home before receiving access to the questionnaire. The principal investigator and a research assistant recruited participants at the school; course and practicum instructors were not involved in recruitment. All 730 eligible students were invited to participate. Participation was voluntary and had no effect on course grades, practicum evaluations, or other academic assessments. Twenty-eight students did not return a signed consent form and therefore were not enrolled. The remaining 702 students returned signed written informed-consent forms and submitted questionnaires. Participants younger than 18 years took the consent documents home before receiving access to the questionnaire. Each minor provided written assent, and a parent or legal guardian provided written permission. The questionnaire QR code was provided only after the research team had verified both required signatures. Data collection was conducted from October to November 2023, after completion of the summer practicum. Three questionnaires were excluded because of incomplete responses, leaving 699 complete records for analysis. All 699 analyzed participants completed the CCFNPS and FNLSS and provided nonblank responses to both open-ended questions. Of these participants, 448 were younger than 18 years and 251 were adults (Fig 1).

**Fig 1:**
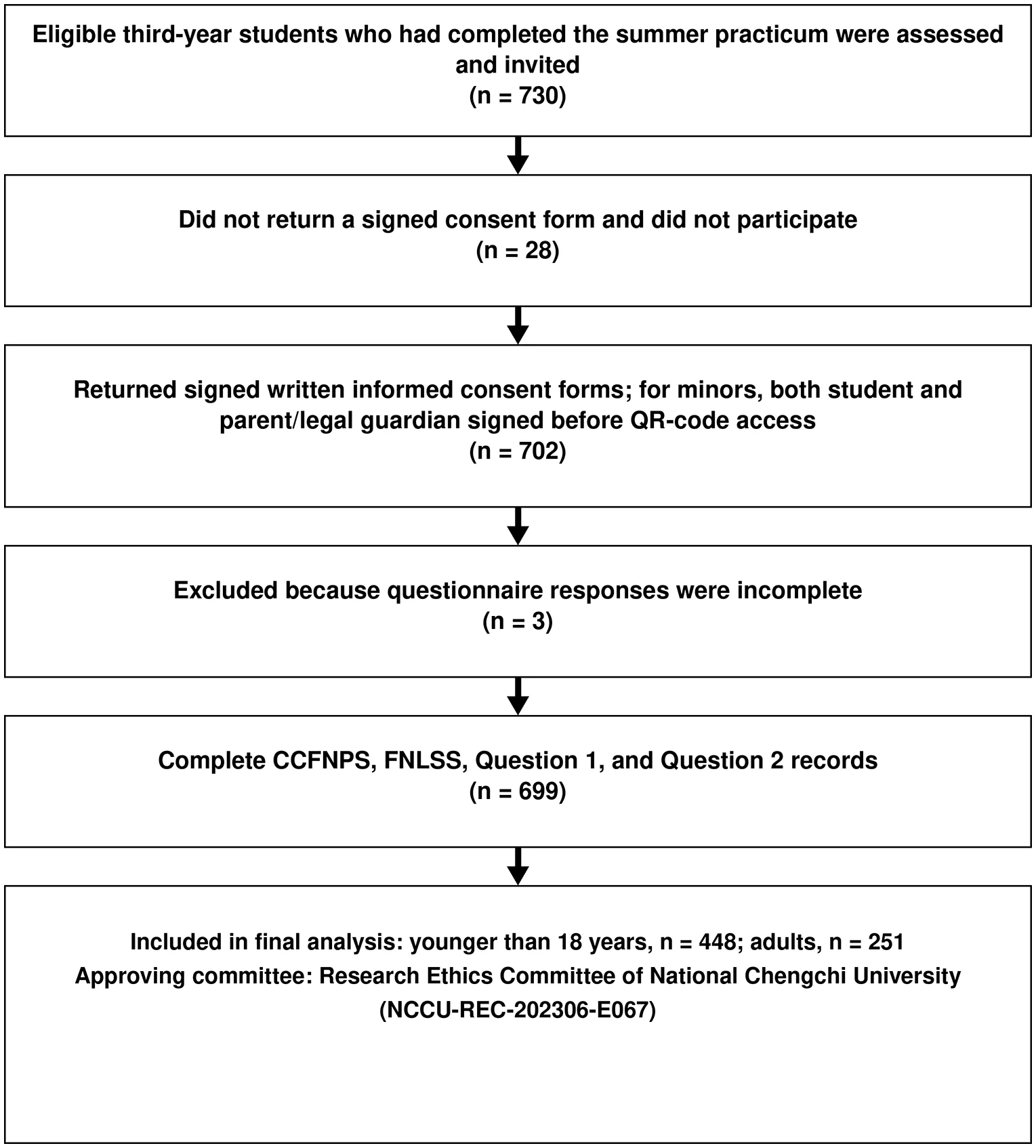
Participant recruitment and data collection procedure.

### Ethics statement

This study was conducted in accordance with applicable ethical standards and was approved by the Research Ethics Committee of National Chengchi University, Taiwan (IRB No. NCCU-REC-202306-E067). The IRB-approved protocol was amended before recruitment to specify third-year students as the target population. Participants were recruited during October-November 2023, after completing the practicum during the summer transition from the second to third year. All participants signed a written informed consent form before accessing the questionnaire. For participants younger than 18 years, both the student and a parent or legal guardian signed the form before it was returned to the research team. The questionnaire QR code was released only after the required signatures had been verified. Participation was voluntary, withdrawal was permitted before submission, and responses were not available to course instructors for grading. Data were de-identified and stored on a password-protected platform accessible only to the research team.

### Measures

#### Participant characteristics.

Background variables included age, sex, admission status (including transfer or reinstatement), paid-work hours per week, self-reported average grade during the previous academic year, psychological-counseling history, and satisfaction with the nursing program. Psychological-counseling history was defined as a self-reported history of having received psychological counseling. The questionnaire did not specify the timing, duration, frequency, indication, or relationship of counseling to the practicum period. Verification against the item-level dataset confirmed that 625 participants reported no counseling history and 74 reported having received counseling. No separate variable assessing learning-support services was included in the questionnaire or dataset; therefore, references to “learning support services” in the previous manuscript version were corrected to “psychological counseling.”

#### Core Competence in Fundamental Nursing Practicum Scale.

The Core Competence in Fundamental Nursing Practicum Scale (CCFNPS), developed by Chang et al. [23], contains 25 items across five domains: communication (3 items), application of the nursing process (6 items), basic biomedical sciences (4 items), nursing skills and ability to perform the care process (7 items), and professional attitude (5 items). All 25 items were administered, and each retained its original instrument number. Students separately rated each competency’s perceived importance (1 = not important to 5 = very important) and their self-rated performance satisfaction (1 = very dissatisfied to 5 = very satisfied). Domain and overall scores were calculated as the means of their constituent items. No CCFNPS item-level data were missing; therefore, complete-case and available-item calculations produced identical scores. For perceived importance, Cronbach’s alpha/McDonald’s omega values were .987/.987 overall, .929/.934 for communication, .977/.978 for application of the nursing process, .950/.952 for basic biomedical sciences, .977/.977 for nursing skills/care-process performance, and .945/.947 for professional attitude. Corresponding alpha/omega values for performance satisfaction were .985/.986 overall, .948/.952, .974/.974, .955/.956, .967/.967, and .933/.934, respectively. Corrected item–total correlations ranged from .779 to .907 for importance and from .741 to .897 for performance satisfaction. Although these coefficients indicate very high internal consistency, they may also reflect overlap or redundancy among similarly worded items and should not, by themselves, be interpreted as evidence of construct validity.

#### Adapted Fundamental Nursing Practicum Learning Stress Questionnaire.

The 22-item Fundamental Nursing Practicum Learning Stress questionnaire (FNLSS) was adapted from stress-related content reported in previous literature [24,25] and was intended to assess classroom learning stress (10 items) and clinical-practicum stress (12 items). However, the original item-generation and selection process, expert-panel composition, item- and scale-level content-validity indices, pilot testing, and formal translation or adaptation procedures could not be reproducibly reconstructed from the available study documentation. Accordingly, the two-domain scoring represents the intended content grouping rather than a measurement structure established in the present sample. Items were rated from 1 (strongly disagree) to 5 (strongly agree). The documented scoring key designated items 7–10, 17–20, and 22 as reverse-scored so that higher scores represented greater reported stress. An item-level audit confirmed that every stored reverse-scored value equaled 6 minus the corresponding original response and that the recalculated subscale and overall means exactly matched the stored scale scores for all participants. These verified recoded values were used in all primary reliability, factor, and stress-score analyses. No FNLSS item-level data were missing; therefore, complete-case scoring was used.

Cronbach’s alpha/McDonald’s omega values were .696/.627 for classroom stress, .790/.744 for clinical-practicum stress, and .847/.774 for overall stress. Corresponding corrected item–total correlations ranged from −.039 to .687, − .098 to .732, and −.066 to .733, respectively. The negative coefficients remained after verification and application of the documented scoring key, raising concerns regarding item functioning and measurement validity. As a coding-direction diagnostic only, analyses of the unreversed raw-item responses produced higher alpha coefficients (.874, .878, and .924, respectively) and uniformly positive corrected item–total correlations. However, these alternative values were not substituted for the prespecified scores because doing so would contradict the documented scoring rule and would not establish construct validity. Maximum-likelihood confirmatory factor analysis (CFA) of the prespecified correlated two-factor structure demonstrated poor fit: χ²(208) = 5861.46, P < .001, CFI = .540, TLI = .481, RMSEA = .197, and SRMR = .205. An ordinal diagonally weighted least-squares sensitivity analysis based on polychoric correlations also demonstrated poor fit: χ²(208) = 5860.92, P < .001, CFI = .790, TLI = .767, RMSEA = .197, and SRMR = .255. Thus, the lack of support for the intended two-factor structure was robust to treating the five-category items as ordinal indicators. The FNLSS is therefore described as an adapted rather than validated questionnaire, and all analyses using its classroom, practicum, and overall scores are treated as exploratory. The complete 22-item instrument and item-level psychometric results are provided in the Supporting Information.

#### Open-ended questions.

The open-ended questions were developed based on literature review [26,27]. Nursing students were asked two open-ended questions to elicit qualitative feedback regarding their learning experiences in the fundamental nursing practicum: (1) “What was your greatest gain during the practicum?” and (2) “What was your greatest difficulty during the practicum?”

### Data analysis

#### Quantitative analysis.

Quantitative analyses were conducted using IBM SPSS Statistics version 27.0 and independently verified using the item-level dataset. Descriptive statistics included frequencies, percentages, means, and standard deviations. Scale scores required complete responses to all constituent items; no CCFNPS or FNLSS item-level data were missing. Internal consistency was assessed using Cronbach’s alpha, McDonald’s omega estimated from a one-factor model, and corrected item–total correlations. The prespecified correlated two-factor FNLSS structure was initially evaluated using maximum-likelihood CFA, with the five-category item responses treated as approximately continuous. Maximum likelihood was retained as the primary estimator because of the large sample and the availability of five response categories. To determine whether the poor model fit depended on this approximation, a sensitivity CFA treated all items as ordinal indicators, estimated polychoric correlations, and used diagonally weighted least squares. The documented reverse-score transformations and stored subscale and overall scores were reproduced record by record from the original item responses. Reliability analyses based on unreversed raw-item coding were performed solely as a coding-direction diagnostic and were not used to redefine the prespecified scale.

For IPA, perceived importance was plotted on the x-axis and self-rated performance satisfaction on the y-axis. Item-weighted sample grand means of 4.679 for importance and 4.503 for performance satisfaction served as the primary crosshairs. Paired importance–performance gaps were tested for each domain and summarized with 95% confidence intervals (CIs). Five thousand bootstrap resamples were used to estimate percentile CIs for both coordinates and the proportion of resamples in which each domain retained its original quadrant after the sample crosshairs were re-estimated. A sensitivity analysis using the scale midpoint of 3.0 as the alternative crosshair was also conducted.

Academic-average categories for inferential analyses were 90–100, 80–89, 70–79, and <70. The 60–69 (n = 122) and <60 (n = 9) categories reported separately in Table 1 were combined into a < 70 group because the < 60 subgroup was too small to provide a stable separate estimate. Exploratory one-way analyses of variance with Scheffé post hoc comparisons examined stress-score differences across academic-average groups. Independent-samples t tests descriptively compared participants with and without a psychological-counseling history. Homogeneity of variance was assessed using median-centered Levene (Brown–Forsythe) tests. Because formal residual-normality tests can be overly sensitive in large samples, residual distributions, skewness, and kurtosis were examined, and Welch ANOVA and Kruskal–Wallis tests were conducted as sensitivity analyses. Welch’s t test was used when the equal-variance assumption was not met. Benjamini–Hochberg false-discovery-rate (FDR) correction was applied across the six omnibus stress comparisons: three academic-average comparisons and three counseling-history comparisons.

**Table 1:** Demographic and academic characteristics of nursing students (N = 699).

Variable	Number (%)	Mean (SD)
Age (years)		17.38 (0.56)
Sex		
Male	57(8.2)	
Female	642(91.8)	
Enrollment status		
No	669 (95.7)	
External transfer admission to the undergraduate program	25(3.6)	
Internal transfer of major to this department	3(0.4)	
Reinstated (resumed studies after a leave of absence)	2(0.2)	
Paid-work hours per week		
No	513(73.4)	
1–8 hours	58(8.3)	
9–16 hours	95(13.6)	
17–24 hours	20(2.9)	
25–32 hours	8(1.1)	
33–40 hours	3(0.4)	
≥41 hours	2(0.3)	
Courses failed (previous academic year)		
All courses passed (No failed courses)	599(85.7)	
Failed 1 course	46(6.6)	
Failed 2 courses	24(3.4)	
Failed 3 courses	15(2.1)	
Failed 4 or more courses	15(2.1)	
Average grade (previous academic year)		
90–100	48(6.9)	
80–89	242(34.6)	
70–79	278(39.8)	
60–69	122(17.5)	
<60	9(1.3)	
Psychological counseling history		
No	625(89.4)	
Yes	74(10.6)	
Interpersonal issues	49	
Emotional problems	39	
Family factors	38	
Academic learning	19	
Interest–career mismatch	3	
Other	3	
Satisfaction with studies (program satisfaction)		
Very satisfied	213(30.5)	
Satisfied	235(33.6)	
Neutral	236(33.8)	
Dissatisfied	10(1.4)	
Very dissatisfied	5(0.7)	

Abbreviation: SD, standard deviation.

Exploratory adjusted ordinary least-squares models included academic-average category, age, sex, paid-work status, psychological-counseling history, program satisfaction, and nonstandard enrollment status, including transfer or reinstatement. Heteroskedasticity-consistent HC3 standard errors were used to address heteroskedasticity but did not account for within-cluster dependence. Values reported as B divided by the outcome standard deviation represent descriptive rescalings of the unstandardized coefficients and are not conventional standardized regression coefficients. FDR correction was applied across the nine adjusted academic-average contrasts. Students were nested within 16 classes and were additionally grouped by three-week rotation, 29 practicum units, 22 teaching-hospital campuses, and 29 clinical instructors. However, participant-level identifiers for class, rotation, instructor, hospital, ward, and practicum unit were not collected. Consequently, intraclass correlation coefficients and cluster-adjusted or multilevel models could not be estimated. If meaningful within-cluster dependence was present, the reported P values may be anti-conservative and the CIs too narrow. All statistical tests were two-sided. Reporting followed the STROBE guidance for cross-sectional observational studies.

#### Supplementary descriptive analysis of open-ended comments.

The two open-ended questions were administered concurrently with the quantitative questionnaire and analyzed as supplementary descriptive comments using thematic content analysis. Although the questions had initially been described as optional, verification of the item-level dataset showed that all 699 participants provided nonblank responses to Question 1, Question 2, and both questions. Consequently, there were no nonrespondents with whom respondent characteristics could be compared. All original responses were written in Chinese.

Two researchers (HWT and SCC) independently reviewed the responses and defined a meaningful response segment as the coding unit. Nonblank entries that did not contain a substantively interpretable idea relevant to the question—including very brief, vague, repetitive, or off-topic responses—were retained in the response denominator but were not assigned to a theme. The researchers inductively developed preliminary codes and a codebook, compared their independently assigned codes, and organized the consensus codes into themes and two broader categories. This process produced 128 segment-to-theme assignments for Question 1 and 89 for Question 2. Participants could contribute more than one meaningful segment, and an individual segment could contribute to more than one theme. Therefore, the summed theme frequencies represent non-mutually-exclusive coding assignments rather than unique participants and should not be interpreted as prevalence estimates.

Initial intercoder percentage agreement before consensus discussion was 92%; Cohen’s kappa was not calculated. Coding disagreements and responses that were ambiguous, difficult to classify, or inconsistent with the predominant themes were discussed by both researchers and, when necessary, examined through expert consultation. Negative and divergent cases were retained rather than excluded or forced into the predominant themes. Final coding decisions were reached through consensus. The codebook, successive coding versions, consensus-meeting records, and decisions regarding ambiguous and negative cases were retained as an audit trail.

Both researchers were bilingual. Selected quotations were translated from Chinese into English, back-translated, and checked against the original responses by both researchers. Translation discrepancies were resolved through discussion. As nursing and nursing-education researchers familiar with fundamental nursing practicums, HWT and SCC recognized that their disciplinary backgrounds, professional roles, and prior expectations could influence theme identification and interpretation. Independent coding, reflexive discussion, expert consultation, examination of negative cases, and maintenance of an audit trail were used to make these potential influences explicit and reduce unexamined interpretive bias.

The supplementary descriptive comments were integrated with the quantitative findings during interpretation by means of a joint display that compared quantitative patterns with related written-response themes. Both convergent and divergent findings were retained rather than forcing agreement between the two sources of data.

## Results

### Participant characteristics

Of the 730 eligible students invited to participate, 28 did not return a signed consent form and were not enrolled. The remaining 702 students returned written informed-consent forms and submitted questionnaires. Three incomplete questionnaires were excluded, leaving 699 participants in the final analysis (95.8% of those invited). Following verification and correction of one age-entry error, the mean age was 17.38 years (SD, 0.56; range, 16–22 years), and 448 participants (64.1%) were younger than 18 years. Most participants were female (n = 642, 91.8%). Regarding enrollment status, 669 (95.7%) reported no transfer or reinstatement, 25 (3.6%) had entered through external transfer, 3 (0.4%) had transferred internally, and 2 (0.3%) had resumed their studies after a leave of absence. Most participants reported no paid employment (n = 513, 73.4%). During the previous academic year, 599 (85.7%) had passed all courses, 46 (6.6%) had failed one course, and 54 (7.7%) had failed two or more courses. The most common previous-academic-year average categories were 70–79 (n = 278, 39.8%) and 80–89 (n = 242, 34.6%). Seventy-four participants (10.6%) reported a history of psychological counseling. Satisfaction with the nursing program was rated as very satisfied by 213 participants (30.5%), satisfied by 235 (33.6%), neutral by 236 (33.8%), dissatisfied by 10 (1.4%), and very dissatisfied by 5 (0.7%) (Table 1).

### Importance-performance analysis by competency domain

Using grand-mean crosshairs (importance = 4.679; satisfaction = 4.503), nursing skills/care process and professional attitude were stably positioned in Quadrant I in 100% of bootstrap samples. Nursing process remained in Quadrant III in 100%. Communication and biomedical sciences lay close to the crosshairs and retained Quadrant I in only 51.3% and 52.1% of bootstrap samples, respectively, indicating classification uncertainty. All importance scores exceeded paired satisfaction scores: communication: gap = 0.164 (95% CI, 0.120–0.207); paired t(698) = 7.42, P < .001; bootstrap retention in Quadrant 1 = 51.3%; nursing process: gap = 0.229 (95% CI, 0.179–0.280); paired t(698) = 8.89, P < .001; bootstrap retention in Quadrant 3 = 100.0%; biomedical sciences: gap = 0.163 (95% CI, 0.123–0.203); paired t(698) = 8.00, P < .001; bootstrap retention in Quadrant 1 = 52.1%; nursing skills/care process: gap = 0.164 (95% CI, 0.131–0.198); paired t(698) = 9.56, P < .001; bootstrap retention in Quadrant 1 = 100.0%; professional attitude: gap = 0.146 (95% CI, 0.111–0.182); paired t(698) = 8.13, P < .001; bootstrap retention in Quadrant 1 = 100.0%. With 3.0 as the alternative crosshair, all domains were high/high (Table 2; Fig 2).

**Table 2:** Core competence by domain during the fundamental nursing practicum (N = 699).

Variable	Perceived importance	Self-rated performance satisfaction	IPA	Importance-performance gap (95% CI)	Bootstrap primary-quadrant retention
Variable	Mean (SD)	Mean (SD)	IPA	Mean difference	%
Core competence in fundamental nursing practicum (CCFNPS)	4.68(0.55)	4.50(0.64)	--		
Communication (CCFNPS)	4.68(0.58)	4.52(0.71)	I	0.164 (0.120-0.207)	Q1: 51.3%
Application of nursing process	4.61(0.66)	4.38(0.78)	III	0.229 (0.179-0.280)	Q3: 100.0%
Basic biomedical science (CCFNPS)	4.68(0.60)	4.52(0.70)	I	0.163 (0.123-0.203)	Q1: 52.1%
Nursing skills and ability to perform a care process (CCFNPS)	4.71(0.56)	4.55(0.64)	I	0.164 (0.131-0.198)	Q1: 100.0%
Professional attitude (CCFNPS)	4.71(0.55)	4.57(0.65)	I	0.146 (0.111-0.182)	Q1: 100.0%

Abbreviations: IPA, importance-performance analysis; SD, standard deviation; CCFNPS, Core Competence in Fundamental Nursing Practicum Scale. The theoretical range is 1–5.

Bootstrap retention is the percentage of 5,000 resamples in the listed quadrant after re-estimating sample crosshairs. All paired gap P values were < .001.

**Fig 2:**
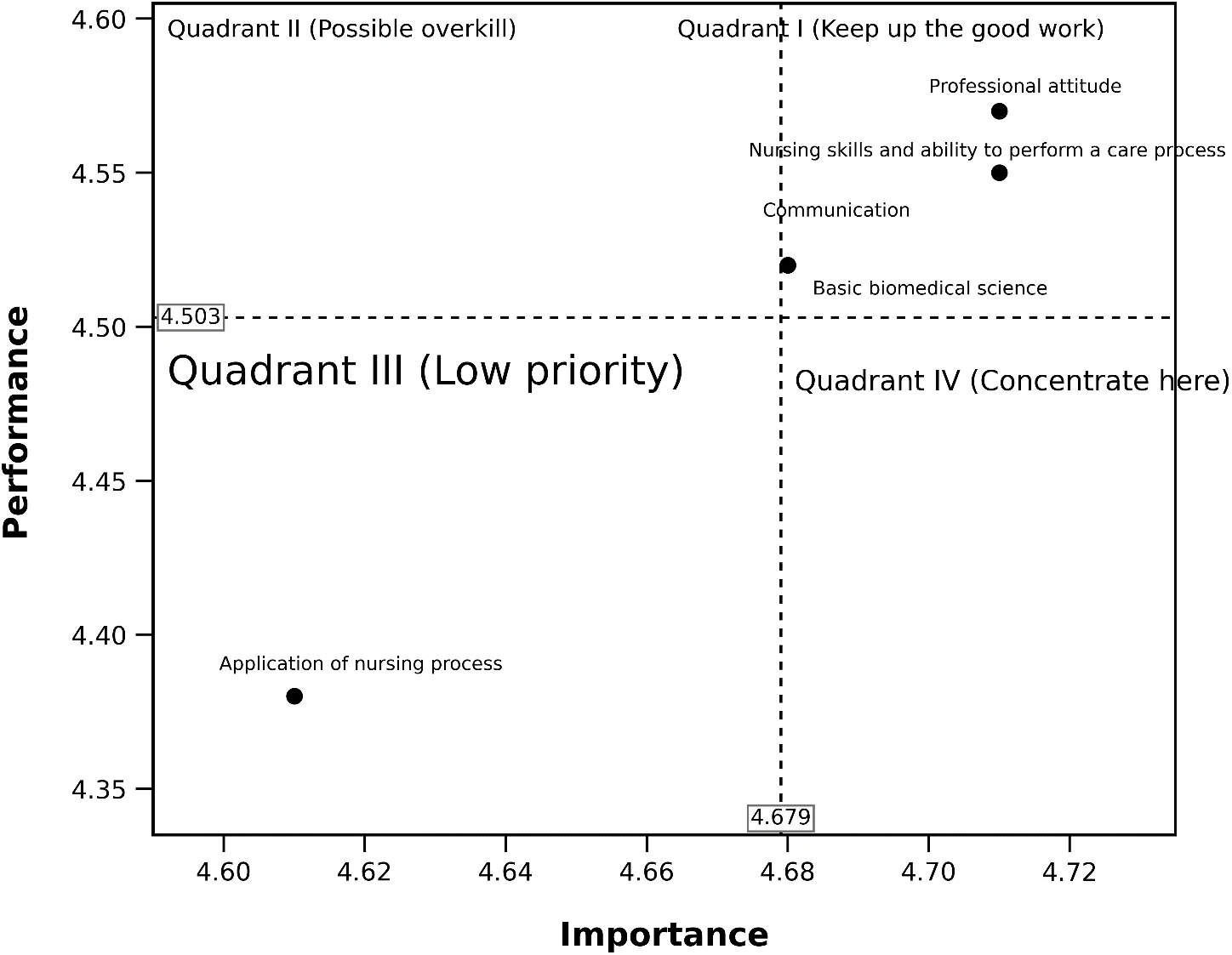
Importance–performance analysis of nursing students’ perceived importance and self-rated performance satisfaction at the competency-domain level during the fundamental nursing practicum.

### Importance-performance analysis by competency item

At item level, 15 items (A1, A2, C1, C2, C4, D1–D5, and E1–E5) were at or above both sample grand means. Ten items (A3, B1–B6, C3, and D6–D7) were below both grand means. Because all item means remained well above the neutral midpoint of 3.0, these groupings represent relative rankings within this sample and should not be interpreted as evidence of low absolute importance, poor observed performance, or a demonstrated curricular priority (Table 3; Fig 3).

**Table 3:** Core competence by item during the fundamental nursing practicum (N = 699).

Variable	Perceived importance	Self-rated performance satisfaction	IPA
Variable	Mean (SD)	Mean (SD)	IPA
Communication (CCFNPS)			
A1 1. Ability to use communication skills to communicate with patients.	4.71(0.59)	4.55(0.72)	I
A2 2. Ability to use communication skills to communicate with caregivers.	4.69(0.61)	4.55(0.72)	I
A3 6. Ability to observe the nonverbal needs of patients.	4.64(0.65)	4.45(0.78)	III
Application of nursing process			
B1 10. Ability to make appropriate nursing diagnoses for patients.	4.60(0.69)	4.38(0.83)	III
B2 11. Ability to make appropriate care plans according to patients’ nursing diagnoses.	4.59(0.72)	4.38(0.82)	III
B3 12. Ability to execute care plans developed for patients.	4.61(0.70)	4.36(0.84)	III
B4 14. Ability to make prompt corrections to care plans for patients per evaluations.	4.61(0.70)	4.36(0.84)	III
B5 13. Ability to evaluate changes to patients after the implementation of care plans.	4.63(0.66)	4.39(0.81)	III
B6 9. Ability to correctly perform physical assessments of patients.	4.62(0.68)	4.40(0.81)	III
Basic biomedical science (CCFNPS)			
C1 16. Ability to understand the side effects of medications used by patients.	4.71(0.62)	4.57(0.70)	I
C2 15. Ability to understand reasons for prescribing specific medications for patients.	4.71(0.61)	4.55(0.73)	I
C3 18. Ability to instruct patients on medication use.	4.63(0.71)	4.44(0.81)	III
C4 17. Ability to understand mechanisms of medication used by patients.	4.68(0.64)	4.51(0.73)	I
Nursing skills and ability to perform a care process (CCFNPS)			
D1 34. Ability to organize data when writing a report.	4.73(0.58)	4.56(0.70)	I
D2 35. Ability to comprehensively consolidate collected information in a written report.	4.73(0.58)	4.56(0.71)	I
D3 33. Ability to search for information.	4.74(0.57)	4.56(0.69)	I
D4 32. Ability to complete required homework or report assignments.	4.73(0.58)	4.58(0.67)	I
D5 23. Ability to operate instruments correctly when performing nursing skills.	4.73(0.59)	4.59(0.67)	I
D6 24. Ability to properly perform nursing skills for patients.	4.65(0.65)	4.47(0.76)	III
D7 22. Ability to properly prepare tools or materials for conducting nursing skills.	4.67(0.63)	4.50(0.73)	III
Professional attitude (CCFNPS)			
E1 29. Ability to cope with frustration.	4.72(0.60)	4.59(0.71)	I
E2 30. Possession of a positive learning attitude.	4.68(0.65)	4.51(0.78)	I
E3 27. Ability to perform emotional self- regulation.	4.68(0.64)	4.53(0.76)	I
E4 31. Ability to perform self-reflections.	4.74(0.56)	4.59(0.71)	I
E5 28. Ability for proper time management after the practicum.	4.75(0.55)	4.62(0.68)	I

Abbreviations: IPA, importance-performance analysis; SD, standard deviation; CCFNPS, Core Competence in Fundamental Nursing Practicum Scale. The theoretical range is 1–5.

**Fig 3:**
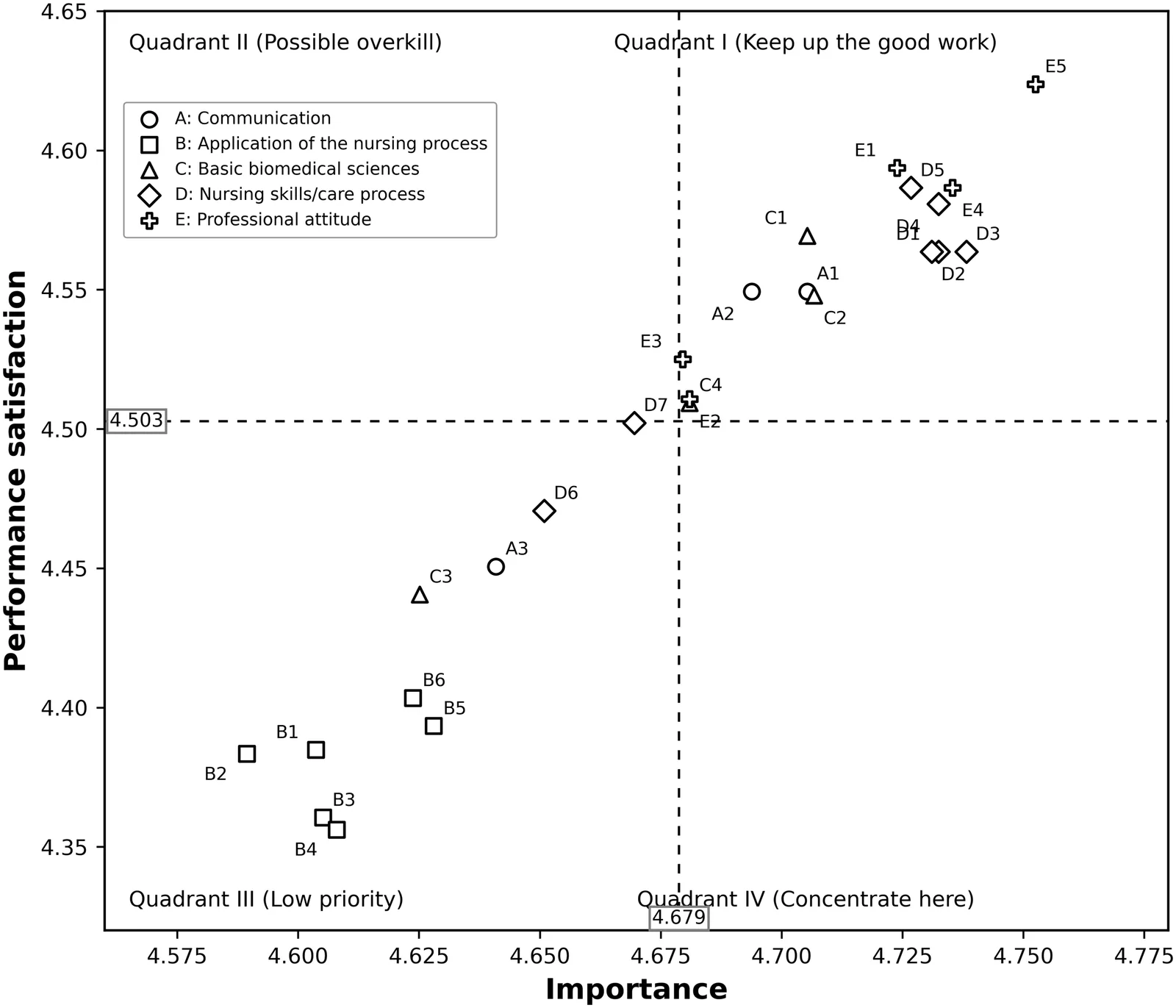
Importance–performance analysis of nursing students’ perceived importance and self-rated performance satisfaction at the individual-item level during the fundamental nursing practicum.

### Exploratory stress-score comparisons by previous-academic-year average

In exploratory analyses using the adapted FNLSS scores, reported stress differed across previous-academic-year average categories for classroom stress, F(3, 695) = 3.91, P = .009, FDR q = .026, partial η² = .017; clinical-practicum stress, F(3, 695) = 3.30, P = .020, q = .040, partial η² = .014; and overall stress, F(3, 695) = 4.19, P = .006, q = .026, partial η² = .018. The homogeneity-of-variance assumption was met for all three outcomes (Levene P = .708, .500, and .172, respectively). Although the residual Shapiro–Wilk tests were statistically significant, residual skewness and kurtosis were modest, and the Welch ANOVA and Kruskal–Wallis sensitivity analyses produced the same overall conclusions. Scheffé post hoc comparisons indicated that the < 70 group reported higher classroom stress than the 80–89 group (P = .032), higher clinical-practicum stress than the 90–100 group (P = .044), and higher overall stress than both the 90–100 (P = .025) and 80–89 groups (P = .041) (Table 4). These findings should be interpreted as exploratory because the intended FNLSS factor structure was not supported and within-cluster dependence could not be modeled.

**Table 4:** Exploratory comparison of adapted FNLSS scores by previous-academic-year average (N = 699).

Variable	Previous-academic-year average	N	Mean (SD)	F (df), exact P, FDR q, partial eta-squared	Scheffe post hoc comparison
Fundamental nursing classroom learning stress	100–90	48	2.81(0.46)	3.91 (3, 695), P = 0.009, q = 0.026, eta-p2 = 0.017	<70> 80-89 (P = .032)
	89–80	242	2.88(0.50)		
	79–70	278	2.94(0.50)		
	<70	131	3.04(0.50)		
					
Fundamental nursing practicum stress	100–90	48	2.39(0.57)	3.30 (3, 695), P = 0.020, q = 0.040, eta-p2 = 0.014	<70> 90-100 (P = .044)
	89–80	242	2.53(0.65)		
	79–70	278	2.57(0.65)		
	<70	131	2.70(0.63)		
					
Overall learning stress	100–90	48	2.58(0.47)	4.19 (3, 695), P = 0.006, q = 0.026, eta-p2 = 0.018	<70> 90-100 (P = .025) and 80–89 (P = .041)
	89–80	242	2.69(0.54)		
	79–70	278	2.74(0.53)		
	<70	131	2.85(0.51)		

*P < 0.05; **P < 0.01; ***P < 0.001. Post hoc comparisons were performed using Scheffe tests.

### Learning stress by psychological-counseling history

In descriptive, exploratory comparisons, none of the stress-score differences between participants without (n = 625) and with (n = 74) a psychological-counseling history remained statistically significant after FDR correction. Mean classroom stress was 2.92 (SD, 0.50) among participants without a counseling history and 3.03 (SD, 0.46) among those with a counseling history, yielding a mean difference of 0.108 (95% CI, −0.013 to 0.228), t(697) = 1.75, P = .080, q = .096, Cohen’s d = 0.216. Corresponding clinical-practicum stress scores were 2.55 (SD, 0.65) and 2.67 (SD, 0.61), respectively, with a mean difference of 0.117 (95% CI, −0.038 to 0.272), t(697) = 1.48, P = .140, q = .140, Cohen’s d = 0.182. Because the equal-variance assumption was not met for overall stress (Levene P = .037), Welch’s t test was used. Mean overall stress was 2.72 (SD, 0.53) without a counseling history and 2.83 (SD, 0.47) with a counseling history, yielding a mean difference of 0.113 (95% CI, −0.003 to 0.229), t(96.86) = 1.93, P = .057, q = .085, Cohen’s d = 0.214 (Table 5). These associations neither demonstrate nor refute counseling effectiveness because counseling was not assigned, its timing and indication were not recorded, baseline stress was unavailable, and the measurement validity of the adapted FNLSS was uncertain.

**Table 5:** Descriptive comparison of adapted FNLSS scores by psychological-counseling history (N = 699).

Outcome	No counseling n, M (SD)	Counseling n, M (SD)	Difference (95% CI)	t (df)	P	FDR q	Cohen d
Classroom stress	625, 2.92 (0.50)	74, 3.03 (0.46)	0.108 (−0.013 to 0.228)	1.75 (697.00)	0.080	0.096	0.216
Practicum stress	625, 2.55 (0.65)	74, 2.67 (0.61)	0.117 (−0.038 to 0.272)	1.48 (697.00)	0.140	0.140	0.182
Overall stress	625, 2.72 (0.53)	74, 2.83 (0.47)	0.113 (−0.003 to 0.229)	1.93 (96.86)	0.057	0.085	0.214

Abbreviations: CI, confidence interval; FDR, false discovery rate; SD, standard deviation. Mean differences are counseling Yes minus No. Welch's t test was used for overall stress because the homogeneity-of-variance test was significant.

### Adjusted models of learning stress

In exploratory covariate-adjusted models using the adapted FNLSS scores (n = 699), participants in the < 70 previous-academic-year average category reported higher classroom stress (B = 0.236, 95% HC3 CI, 0.082–0.390; FDR q = .012), clinical-practicum stress (B = 0.286, 95% HC3 CI, 0.089–0.484; q = .013), and overall stress (B = 0.263, 95% HC3 CI, 0.106–0.421; q = .010) than participants in the 90–100 reference category. The contrasts between the 70–79 and 90–100 categories did not remain statistically significant after FDR correction. After covariate adjustment, psychological-counseling history was not independently associated with classroom stress (P = .169), clinical-practicum stress (P = .252), or overall stress (P = .161). Adjusted R² values were .045, .055, and .062 for the classroom, clinical-practicum, and overall stress models, respectively (Table 6). These estimates should be interpreted cautiously because the prespecified FNLSS factor structure was not supported and HC3 standard errors did not account for potential within-class, rotation, practicum-unit, site, or instructor dependence.

**Table 6:** Exploratory covariate-adjusted linear models of adapted FNLSS scores (n = 699).

Outcome	Predictor	B	95% HC3 CI	B / outcome SD	P	FDR q*
Classroom	Age (years)	−0.009	−0.077 to 0.059	−0.018	.797	
Classroom	Female (vs male)	−0.055	−0.211 to 0.102	−0.109	.494	
Classroom	Paid work (yes vs no)	0.008	−0.076 to 0.093	0.017	.844	
Classroom	Counseling (yes vs no)	0.083	−0.035 to 0.201	0.166	.169	
Classroom	Transfer/reinstatement (vs none)	−0.003	−0.198 to 0.193	−0.005	.979	
Classroom	Grade 80–89 (vs 90–100)	0.090	−0.054 to 0.233	0.180	.220	0.220
Classroom	Grade 70–79 (vs 90–100)	0.143	0.002 to 0.284	0.286	.047	0.085
Classroom	Grade <70 (vs 90–100)	0.236	0.082 to 0.390	0.472	.003	0.012
Classroom	Satisfied (vs very satisfied)	0.000	−0.101 to 0.101	0.000	.997	
Classroom	Neutral (vs very satisfied)	0.172	0.076 to 0.268	0.345	<.001	
Classroom	Dissatisfied (vs very satisfied)	0.406	0.124 to 0.687	0.812	.005	
Classroom	Very dissatisfied (vs very satisfied)	0.542	0.181 to 0.904	1.086	.003	
Practicum	Age (years)	−0.034	−0.124 to 0.056	−0.052	.463	
Practicum	Female (vs male)	−0.206	−0.360 to −0.053	−0.320	.008	
Practicum	Paid work (yes vs no)	0.103	−0.002 to 0.208	0.160	.055	
Practicum	Counseling (yes vs no)	0.089	−0.063 to 0.240	0.137	.252	
Practicum	Transfer/reinstatement (vs none)	−0.104	−0.336 to 0.128	−0.162	.379	
Practicum	Grade 80–89 (vs 90–100)	0.153	−0.032 to 0.338	0.238	.104	0.117
Practicum	Grade 70–79 (vs 90–100)	0.174	−0.007 to 0.355	0.270	.060	0.090
Practicum	Grade <70 (vs 90–100)	0.286	0.089 to 0.484	0.444	.004	0.013
Practicum	Satisfied (vs very satisfied)	0.037	−0.088 to 0.163	0.058	.560	
Practicum	Neutral (vs very satisfied)	0.227	0.104 to 0.349	0.352	<.001	
Practicum	Dissatisfied (vs very satisfied)	0.603	0.427 to 0.779	0.936	<.001	
Practicum	Very dissatisfied (vs very satisfied)	0.565	0.335 to 0.795	0.877	<.001	
Overall	Age (years)	−0.022	−0.092 to 0.047	−0.042	.530	
Overall	Female (vs male)	−0.137	−0.268 to −0.007	−0.260	.039	
Overall	Paid work (yes vs no)	0.060	−0.027 to 0.147	0.114	.178	
Overall	Counseling (yes vs no)	0.086	−0.034 to 0.206	0.163	.161	
Overall	Transfer/reinstatement (vs none)	−0.058	−0.234 to 0.118	−0.110	.518	
Overall	Grade 80–89 (vs 90–100)	0.124	−0.024 to 0.272	0.236	.100	0.117
Overall	Grade 70–79 (vs 90–100)	0.160	0.015 to 0.304	0.303	.030	0.068
Overall	Grade <70 (vs 90–100)	0.263	0.106 to 0.421	0.499	.001	0.010
Overall	Satisfied (vs very satisfied)	0.020	−0.084 to 0.125	0.039	.702	
Overall	Neutral (vs very satisfied)	0.202	0.101 to 0.303	0.383	<.001	
Overall	Dissatisfied (vs very satisfied)	0.513	0.311 to 0.715	0.973	<.001	
Overall	Very dissatisfied (vs very satisfied)	0.555	0.298 to 0.811	1.051	<.001	

*Benjamini-Hochberg FDR q values are shown for the nine GPA contrasts. HC3 robust standard errors addressed heteroskedasticity but did not correct for within-class or within-practicum dependence. Clustering could not be modeled because participant-level class, instructor, rotation, and site identifiers were unavailable. B/outcome SD is a descriptive rescaling of the unstandardized coefficient and is not a conventional standardized regression coefficient.

### Supplementary descriptive findings from open-ended comments

All 699 participants provided nonblank responses to both Question 1 and Question 2. However, many entries were very brief, vague, repetitive, off-topic, or otherwise lacked a substantively interpretable idea relevant to the question. These entries were retained in the response denominator but were not assigned to a theme. The substantively meaningful material produced 128 non-mutually-exclusive segment-to-theme assignments for perceived practicum gains and 89 for practicum difficulties, yielding 217 assignments in total. Initial intercoder percentage agreement before consensus discussion was 92%; Cohen’s kappa was not calculated. Coding disagreements were resolved through discussion and consensus, with expert consultation used when additional clarification was required. These frequencies represent coding assignments rather than unique participants or prevalence estimates (Table 7). The relationships between the quantitative findings and the supplementary written-response themes, together with their interpretive boundaries, are presented in the joint display (Table 8).

**Table 7:** Themes from students’ reflections during the fundamental nursing practicum.

Themes	Sample Quotes	Theme assignments, n
1. Gains from the Fundamental Nursing Practicum		
1.1 Improvement in technical and theoretical competence	“I became more skilled and accurate in performing procedures.” / “Now I understand how to educate real patients and recognize more medications.”	28
1.2 Application of the nursing process	“I learned to identify individualized nursing problems and provide appropriate interventions.” / “I can proactively discover and solve patient problems.”	20
1.3 Enhancement of core competencies (critical thinking, communication, collaboration, caring)	“I learned to think critically and plan before acting.” / “I can communicate better with patients and families and cooperate with classmates.”	26
1.4 Bridging theory and clinical practice	“Everything from the textbook came alive before my eyes.” / “I finally understood the real difference between classroom and clinical nursing.”	18
1.5 Strengthened professional identity	“I now understand what nurses really do and feel more motivated to continue in this profession.”	14
1.6 Personal growth and self-affirmation	“I became more patient and responsible.” / “My confidence increased, and I can handle pressure better.”	22
2. Difficulties encountered during the Fundamental Nursing Practicum		
2.1 Lack of technical and theoretical knowledge	“I was unfamiliar with some procedures and medical terms.” / “I had difficulty understanding pharmacology and translating doctors’ orders.”	24
2.2 Difficulty in writing clinical assignments	“Writing nursing care plans was hard.” / “I didn’t know how to design appropriate health education for the case.”	15
2.3 Insufficient core competence (communication, teamwork, problem-solving)	“I found it hard to communicate with patients or get subjective data.” / “I didn’t know how to solve problems by myself.”	20
2.4 Stress and anxiety	“I was too nervous during procedures, afraid of making mistakes.” / “I couldn’t handle the emotional stress when patients passed away.”	18
2.5 Interpersonal challenges	“It was hard to get along with senior nurses.” / “Sometimes I couldn’t understand their instructions quickly.”	12

N indicates the number of segment-to-theme assignments. Assignments were not mutually exclusive. The displayed counts sum to 128 assignments for perceived gains and 89 for difficulties (217 total) and must not be interpreted as 217 unique respondents or as prevalence estimates.

**Table 8:** Joint display of quantitative patterns and supplementary written-response themes.

Quantitative pattern	Related written-response theme	Integration	Interpretive boundary
Four domains above both sample grand means	Communication, technical/theoretical competence, and professional identity were described as gains	Convergence	Both strands are self-reported; no objective performance inference
Nursing process below both sample grand means but high in absolute terms	Care-plan writing and theory application were described as difficulties	Contextual convergence	Comments are self-selected and do not make Quadrant III a primary priority
Lower prior grade categories associated with slightly higher stress	Stress and anxiety were described as difficulties	Convergence	Cross-sectional data do not establish direction or mechanism
No significant unadjusted or adjusted association with counseling history	No theme evaluated counseling effectiveness	Divergence/no corroboration	Counseling exposure details and confounding by indication were not measured

## Discussion

Students rated the absolute importance of all five competency domains highly and reported high performance satisfaction. Four domains were at or above the sample grand means on both dimensions. Similar studies have shown that nursing students generally report favorable perceived competency while ratings vary with clinical placement, supervision, learning resources, and self-efficacy [13,17]. Nevertheless, the present scores represent perceptions and satisfaction rather than direct assessments of clinical competence, observed performance, or safe practice. The concentration of ratings near the upper end of the scale also suggests a possible ceiling effect and may partly reflect social desirability, limited early-practicum experience, or perceived faculty expectations.

The application-of-the-nursing-process domain fell below both sample-derived grand means but remained highly rated in absolute terms. Its placement in Quadrant III therefore indicates a lower relative position within an otherwise highly rated set of competencies rather than low educational importance or evidence of a competency deficit. This distinction is important because IPA classifications depend on the selected crosshairs and are intended to support relative rather than absolute prioritization [18,19]. Bootstrap results further qualified the domain-level interpretation. Communication and basic biomedical sciences retained their Quadrant I classifications in only 51.3% and 52.1% of resamples, respectively. In contrast, nursing skills/care-process performance and professional attitude remained in Quadrant I in 100% of resamples, whereas application of the nursing process remained in Quadrant III in 100%. Moreover, when the scale midpoint of 3.0 replaced the sample-derived grand means, all five domains were classified as high importance/high performance satisfaction. Therefore, the Quadrant I classifications of communication and basic biomedical sciences were not highly stable, and all IPA placements should be interpreted as crosshair-dependent descriptive patterns rather than definitive educational priorities [18,19,28].

The supplementary comments regarding care-plan preparation and the application of theory to practice align with qualitative studies describing the challenges nursing students experience when transferring classroom knowledge to clinical situations [26,27]. Educational strategies such as simulation, case-based discussion, guided reflection, and mentorship are consistent with experiential approaches to developing clinical reasoning [29]. However, the effectiveness of these strategies was not evaluated in the present study and should be examined prospectively.

Exploratory analyses using the adapted FNLSS scores indicated higher reported stress among students in lower previous-academic-year average categories, although the omnibus effect sizes were small. After covariate adjustment and FDR correction, participants in the < 70 category reported higher classroom, clinical-practicum, and overall stress scores than those in the 90–100 reference category. However, these statistical associations do not demonstrate that the intended classroom and clinical-practicum stress constructs were validly measured in this sample. Both maximum-likelihood and ordinal diagonally weighted least-squares CFA showed poor model fit, several corrected item–total correlations remained negative after record-level verification of the documented reverse-scoring procedure, and the original instrument-development documentation was incomplete. Although the unreversed raw-item coding produced stronger internal-consistency estimates, replacing the documented scoring key on this data-driven basis would not constitute a defensible validation strategy.

The absence of participant-level cluster identifiers also prevented adjustment for dependence within classes, rotations, practicum units, teaching-hospital campuses, and clinical instructors; the use of HC3 standard errors did not address this source of dependence. Consequently, the reported P values may be anti-conservative and the confidence intervals too narrow. The observed pattern is broadly consistent with evidence linking academic and clinical stressors to nursing students’ learning experiences, perceived competence, and academic engagement [16,20–22], but it should be regarded as hypothesis-generating [30]. Furthermore, the cross-sectional design cannot determine whether lower academic achievement preceded greater reported stress, whether stress adversely affected academic performance, or whether both were influenced by other individual or learning-environment factors.

Psychological-counseling history was not independently associated with any adapted FNLSS score after covariate adjustment. This lack of association should not be interpreted as evidence that psychological counseling was either effective or ineffective. Counseling was not randomly assigned, and information regarding its timing, duration, intensity, indication, adherence, and referral pathway was unavailable. Baseline stress was not measured, and the FNLSS measurement model was not supported in this sample. Moreover, students who had received counseling may have experienced greater pre-existing distress or differed from those without a counseling history in other psychosocial characteristics. Previous studies indicate that nursing students’ clinical stress is associated with multiple personal and learning-environment factors [20–22]. Residual confounding and confounding by indication therefore remain plausible, and the counseling analysis should be interpreted as descriptive and associational rather than evaluative.

The supplementary written comments partially converged with the quantitative findings. Participants described perceived gains in communication and nursing skills while also reporting difficulties involving nursing-process assignments, theory–practice integration, and stress [31]. Previous qualitative studies have similarly identified theory–practice integration, clinical workload, unfamiliar clinical environments, and interpersonal demands as salient features of nursing students’ practicum experiences [26,27]. Although all 699 participants provided nonblank responses to both open-ended questions, only substantively meaningful response segments were assigned to themes. Brief, vague, repetitive, or off-topic entries remained in the response denominator but did not generate a coding assignment. Divergent and negative comments were retained, and the 217 non-mutually-exclusive segment-to-theme assignments were not interpreted as representing 217 unique respondents. Accordingly, these supplementary comments provide context for the survey findings but do not quantify the prevalence of particular experiences or causally explain the observed quantitative patterns.

### Strengths and limitations

This study had several strengths, including a large attempted-census sample, paired ratings of perceived importance and self-rated performance satisfaction, bootstrap assessment of IPA classification stability, and transparent reporting of the supplementary open-ended responses. Nevertheless, several limitations should be considered. First, the cross-sectional design precludes causal or temporal conclusions. Second, all measures were self-reported and cannot establish objectively observed competence, clinical performance, or patient safety. The high CCFNPS scores also suggest a possible ceiling effect. Moreover, the CCFNPS alpha and omega coefficients approached .99, which may reflect overlap or redundancy among similarly worded items in addition to high internal consistency. Third, the young, predominantly female cohort was recruited from a single five-year nursing program, limiting generalizability to other institutions, educational systems, and student populations. Students were nested within 16 classes and were additionally grouped into three-week rotations across 29 practicum units at 22 teaching-hospital campuses, with 29 unique clinical instructors providing supervision. However, participant-level identifiers for class, instructor, hospital, ward, rotation, and practicum unit were not collected. Intraclass correlation coefficients and cluster-adjusted or multilevel estimates therefore could not be calculated. HC3 robust standard errors addressed heteroskedasticity but not within-cluster dependence; consequently, the precision of the exploratory stress estimates may be overstated. Fourth, IPA placement depended on the selected crosshairs [18,19]. Communication and basic biomedical sciences retained their Quadrant I classifications in only 51.3% and 52.1% of bootstrap samples, respectively, whereas use of the scale midpoint classified all domains as high importance/high performance satisfaction. The resulting IPA pattern was therefore crosshair-dependent and should not be interpreted as a definitive educational-priority ranking. Fifth, the adapted FNLSS demonstrated mixed internal consistency, negative corrected item–total correlations after verified application of the documented reverse-scoring procedure, and very poor fit of the prespecified two-factor structure under both maximum-likelihood and ordinal diagonally weighted least-squares estimation. Complete historical documentation of instrument development, content-validity assessment, pilot testing, and language adaptation was also unavailable for verification. Accordingly, analyses using the FNLSS scores should be interpreted as exploratory and hypothesis-generating rather than confirmatory. Sixth, the residual distributions departed from normality. HC3 robust standard errors and nonparametric sensitivity analyses addressed some distributional concerns but could not resolve the measurement and clustering limitations. Seventh, psychological-counseling history lacked information regarding timing, duration, intensity, indication, and content and therefore could not be used to evaluate counseling effectiveness. Finally, theme frequencies represented non-mutually-exclusive coding assignments rather than prevalence estimates, and the brief questionnaire responses did not provide the depth obtainable from individual qualitative interviews.

## Conclusions

Students reported high absolute levels of perceived importance and self-rated performance satisfaction across all fundamental nursing practicum competency domains. Perceived-importance ratings exceeded performance-satisfaction ratings; however, IPA quadrant stability varied across domains, and the classification pattern changed when the scale midpoint replaced the sample-derived crosshairs. Exploratory analyses using the adapted FNLSS scores indicated higher reported stress among students in the < 70 previous-academic-year average category. These associations require cautious interpretation because the prespecified FNLSS factor structure was not supported, several corrected item–total correlations were negative, and within-cluster dependence could not be modeled. Comparisons based on psychological-counseling history were descriptive and did not evaluate counseling effectiveness. Supplementary written comments provided context for the quantitative findings but did not establish the prevalence, directionality, or causal mechanisms of students’ experiences. Future multicenter longitudinal studies should incorporate validated stress measures, objective assessments of clinical performance, participant-level cluster identifiers, clustering-aware statistical analyses, and prospectively evaluated educational and supportive interventions.
